# Recent Progress of Ion Implantation Technique in GaN-Based Electronic Devices

**DOI:** 10.3390/mi16090999

**Published:** 2025-08-29

**Authors:** Hao Lu, Xiaorun Hao, Yichi Zhang, Ling Yang, Bin Hou, Meng Zhang, Mei Wu, Xiaohua Ma, Yue Hao

**Affiliations:** School of Microelectronics, Xidian University, Xi’an 710071, China

**Keywords:** gallium nitride, RF device, fabrication process, ion implantation, HEMT

## Abstract

Gallium nitride (GaN) offers exceptional material properties, making it indispensable in communications, defense, and power electronics. With high electron mobility and robust thermal conductivity, GaN-based devices excel in high-frequency, high-power applications. They are vital in wireless communication systems, radar, electronic warfare, and power electronics systems, offering superior performance, efficiency, and reliability. Further research is crucial for optimizing GaN-based devices performance and expanding their applications, driving innovation across industries. The application of ion implantation technology in GaN-based devices is a key process that can be used to improve device performance and characteristics, which enables process aspects such as electrical isolation, ion implantation for ohmic contacts, threshold voltage regulation, and terminal design. In this paper, we will focus on reviewing the principles and issues of the ion implantation process in GaN-based device preparation. This work aims to serve as a guide for ion implantation in future GaN-based devices.

## 1. Introduction

Gallium nitride (GaN) has emerged as a pivotal material in the realm of electronic devices, particularly in communication technologies. GaN excels in high-frequency and high-power devices due to its superior chemical and physical properties. With a wide bandgap of 3.4 eV, much higher than silicon’s 1.1 eV, it can withstand a higher breakdown electric field of approximately 3.3 MV/cm, enabling operation at higher voltages to boost power density. Additionally, its high electron mobility allows faster electron movement, supporting high-frequency switching and reducing energy loss. Moreover, its good thermal conductivity facilitates rapid heat dissipation, and its high-temperature stability enables it to maintain performance in high-temperature environments, making it ideal for high-power scenarios and extending device lifespan. L. Yang et al. [1] recently developed an AlGaN/GaN/AlN: Fe heterostructure that synergistically enhances quantum well carrier confinement while improving thermal management, achieving superior power handling capabilities in a field-plate-free configuration. This architectural innovation directly addresses the critical requirements of modern 5G wireless infrastructure, where GaN-based devices play a pivotal role in enabling high-power millimeter-wave operations for multi-gigabit data transmission, extended network coverage, and enhanced signal integrity. In addition, GaN technology is contributing to the development of radar systems, satellite communications, and livelihood applications. This technological viability stems fundamentally from the material’s exceptional tolerance to simultaneous thermal, radiative, and high-power density stresses—a triad of stressors that conventionally degrade semiconductor performance [2]. Recent advances in GaN-on-Si HEMT technology, as comprehensively reviewed by H. Lu et al. [3], reveal transformative progress in RF device engineering through synergistic innovations spanning gate structure nano-fabrication, polarization-optimized epitaxial design, and nonlinear behavioral modeling. These developments collectively address the critical 5G implementation challenges of balancing millimeter-wave power efficiency with signal linearity, while advancing cost-effective manufacturing paradigms that maintain quantum confinement advantages at scaled nodes. Ion implantation enables submicron-precision doping in GaN device fabrication, overcoming the inherent challenges of defect-tolerant doping in wide-bandgap semiconductors. By spatially modulating ion species through masked implantation and subsequent rapid thermal annealing protocols, this technique achieves three-dimensional carrier profile engineering critical for both lateral HEMT channel confinement and vertical junction termination. The resultant doping-defect interplay directly governs crucial performance metrics including Schottky barrier uniformity, buffer leakage suppression, and dynamic threshold voltage stability—parameters fundamentally limiting GaN reliability in high-power switching and RF amplification regimes.

Furthermore, ion implantation facilitates the formation of highly localized dopant profiles, enabling the creation of intricate device architectures with enhanced performance and reliability. By carefully calibrating ion energy and dosage, researchers can achieve precise doping profiles tailored to the requirements of various GaN-based applications. Moreover, ion implantation offers the advantages of scalability and reproducibility, making it the preferred choice for the mass production of GaN devices.

Given the importance of GaN-based devices outlined above, GaN technology represents a cornerstone in modern communication systems, offering unparalleled performance and versatility. Ion implantation also enables precise modification of photonic materials’ refractive index and optoelectronic properties, supporting high-density, high-performance integration of photonic integrated circuits [4]. Ion implantation serves as a critical enabler in the fabrication process of GaN devices, empowering engineers to harness the full potential of this remarkable semiconductor material. By delving into the intricacies of ion implantation, researchers can unlock new avenues for innovation and drive the advancement of GaN-based technologies across diverse applications. Therefore, this paper conducts an in-depth discussion on the application of ion implantation in different types of GaN devices, focusing primarily on the mechanisms and impacts of nitrogen ions (N), fluorine ions (F), silicon ions (Si) and argon ions (Ar) in GaN devices. Through this research review of ion implantation technologies, we aim to comprehensively understand their application prospects and potential contributions in GaN devices.

## 2. Electrical Isolation of Ion Implantation in GaN Devices

GaN is widely utilized in high-power and high-frequency electronic devices due to its excellent electronic properties. However, ensuring electrical isolation of GaN devices is crucial for maintaining device performance and stability in practical applications. N ion implantation is a commonly employed technology for electrical isolation and has been extensively studied and applied in GaN devices [5]. Nitrogen ions, with high electronegativity and reactivity, collide with Ga-N covalent bonds in the GaN lattice upon implantation, causing bond breakage and the formation of dangling bonds, which act as carrier traps. Meanwhile, they disrupt the 1:1 Ga:N stoichiometric ratio of GaN, leading to local nitrogen enrichment and the generation of interstitial nitrogen atoms, which induce lattice distortion and scatter carrier transport. Additionally, N^+^ cannot serve as an effective doping source and thus avoids forming conductive channels. Ultimately, a high-resistance region is formed through carrier trapping by defects, carrier scattering, and conduction path blocking, thereby achieving electrical isolation. This section will review the advantages of N ion implantation in electrical isolation for GaN devices, along with current research state and methods.

N ion implantation offers multiple advantages for achieving electrical isolation in GaN devices. Firstly, it is a planar process that avoids sidewall etching damage associated with conventional mesa top etching. For instance, H. Lu et al. [6] proposed in 2021 that AlN/GaN/InGaN CC-HEMTs prepared using a planar process exhibit near-ideal subthreshold swing (SS), a large gate voltage swing (GVS), and consistent values of both the current gain cut-off frequency (*f_T_*) and maximum oscillation frequency (*f_max_*) over a 4-V gate bias range. Secondly, N ions can improve subthreshold characteristics in AlN/GaN/InGaN CC-HEMT devices. Studies have shown that these devices achieve sub-60 mV/dec subthreshold swing and excellent Q through N ion injection. In addition, the effect of N ions is demonstrated by enhanced hot carrier transport with shorter gate lengths, improving the transverse electric field under the gate [7]. Furthermore, compared to alloy contacts, no surface roughness was observed due to N ion injection, which enables electrical isolation without the need for an additional isolation layer, as proposed by F. Recht in 2006 [8].

In N-polar GaN, current blocking layer (CBL) is typically achieved by injecting magnesium ions (Mg^2+^), which diffuse into the channel layer at high temperatures, resulting in an uncontrollable change in the threshold voltage. S. Rajabi et al. [9] (2019) developed a CBL by introducing nitrogen ions into the nitrogen-polar GaN material. This approach ensures that the current can only flow through the apertures of the device, effectively preventing the diffusion of Mg ions. H. Yu et al. [10] proposed that forming isolation regions of samples undergo three—step N ion implantation with specified energies/doses enables high resistivity isolation while maintaining a low defect density. This was achieved due to the low heat-treatment temperature of N ions after injection. TRIM simulates N ion, interstitial/vacancy profiles, indicating ~1000 theoretical carrier removals per N ion, but dynamic annealing—induced defect annihilation lowers experimental rates and causes overestimated defect densities in TRIM results.

The subsequent study further refined this research. Z. Wang et al. [11] (2023) have gained a profound understanding of the influence of high temperature on device performance by investigating the electrical properties of isolated AlGaN/GaN heterojunctions with N implantation at 300 °C. The reduction in leakage current is attributed to the dynamic annealing effect during high-temperature injection, which promotes the reduction of shallow energy level formation. The key conclusion of this study is that the implantation isolation procedure should be conducted after a temperature exceeding 450 °C to achieve lower leakage current. Several combinations of energy and dose were simulated and implemented via TRIM, which form a nitrogen-doped box profile with a bulk concentration of 1.6 × 10^19^ cm^−3^ over a depth of 0.6 μm. Consequently, valuable insights can be obtained for the fabrication, reliability, and bulk production of GaN-based photonics and electronic devices.

High-dose N implantation introduces a high concentration of dopants into GaN devices, which can enhance both isolated leakage suppression and voltage withstand characteristics through two primary mechanisms: (1) optimizing current distribution by appropriately reducing source resistance, or (2) maintaining source resistance stability while enabling partial electron transport from the top GaN channel to the bottom heterojunction region via quantum tunneling or 2DEG barrier penetration under strong electric fields [12]. Nitrogen ion implantation has been shown to enhance device insulation by creating the optimal concentration of electron-hole pairs in GaN and disrupting the lattice structure [13]. H. Yu et al. [14] (2022) proposed that by using low-dose and high-energy N ion injection, lateral penetration of ions can be induced at the sides of AlGaN/AlN/GaN HEMTs. This lateral penetration can cause damage at the sides of HEMTs and lead to the formation of parasitic channels. As a result, the effective width of the isolated HEMTs is reduced by approximately 0.5 μm, while the electrical characteristics of the parasitic channels are altered, manifesting as low on-state conductance, reduced gate current, and a more positive threshold voltage.

In 2023, C. Shi et al. [12] proposed that by introducing N ions, the double channel with graded barrier high electron mobility transistor (DCGB-HEMT) structure effectively diminishes the electric field peak at the gate edge, thereby enhancing voltage withstand characteristics. Moreover, the introduction of N ions contributes to the formation of a three-dimensional electron gas (3DEG) in the bottom heterostructure, effectively suppressing leakage induced by the iron-doped GaN buffer layer and improving the voltage withstand and leakage performance of the DCGB-HEMT.

These advancements in the fabrication process have significantly enhanced the thermal and electrical stability of GaN devices under elevated operating temperatures and electric fields. Nevertheless, further investigations are required to elucidate the stability mechanisms of shallow traps and ensure reliable high-frequency operation.

## 3. Effect of Ion Implantation on Ohmic Contact

The role of Si ion implantation in the low-resistance ohmic contact of GaN devices is an important research area. As a Group Ⅳ element, Si tends to exhibit donor properties in GaN and can provide free electrons. After Si ion implantation, its atoms combine with the GaN lattice, and through chemical electron transfer, the carrier concentration in the material is increased. This makes it easier for current to conduct in the contact area, thereby effectively reducing the ohmic contact resistance [15]. The formation of this doped region can improve the quality of the contact interface and reduce the interface resistance, thereby reducing the on-resistance of the device and improving the working efficiency of the device. Si ion implantation can also control the surface morphology and crystal quality of GaN devices. Through the annealing treatment after Si ion implantation, the GaN surface can form a uniform and flat structure, reduce surface defects and impurities, and improve the crystal quality, thus improving the performance stability and reliability of the device [16]. Research demonstrates that Si ion implantation effectively modulates the interface trap density and energy band characteristics at the GaN/metal interface, optimizing low-resistance ohmic contact formation, improving contact stability and reliability, and ultimately enhancing overall device performance [17].

The functional modes of silicon ion implantation can be systematically classified into two primary categories based on their mechanisms of action [15,16,17]: (1) modulating electrical conductivity through controlled carrier manipulation and (2) minimizing contact resistance via localized doping effects. These two distinct approaches will be systematically analyzed in the subsequent sections.

### 3.1. Modulation of Electrical Conductivity Using Ion Implantation Technique

Back in 2007, K. Nomoto et al. [18] significantly enhanced device performance through ion implantation. Si ions were implanted into GaN/AlGaN/GaN HEMTs’ source/drain regions at room temperature with energies of 30 keV and 80 keV and an effective dose of 1.0 × 10^15^ cm^−2^, followed by annealing at 1200 °C for 2 min in N_2_ ambient, resulting in contact resistances of 0.08 Ω·mm (30 keV) and 0.1 Ω·mm (80 keV). The Si concentration varies with depth, with the peak position at 80 keV coinciding with the heterointerface. The on-state resistance exhibited a significant decrease from 26.2 to 4.3 Ω·mm, whereas the saturation drain current and maximum transconductance demonstrated marked improvements, with values increasing from 284 to 723 mA/mm and 48 to 147 mS/mm, respectively. Subsequently, in 2012, K. Nomoto et al. [19] showed that silicon ion implantation in GaN devices enables high doping in n-type layers, enhanced electron mobility, and precise modulation of carrier density profiles, with ion fluence variation allowing controllable adjustment of key parameters for performance improvements. Using 30 keV Si ions at effective doses of 1 × 10^14^ to 2 × 10^15^ cm^−2^ implanted into undoped GaN and n-type Al_0.25_Ga_0.75_N/GaN, followed by 1250 °C annealing in N_2_ with a Si_3_N_4_ cap, they achieved notable results. SRIM simulations indicated peak Si concentrations near the Si_3_N_4_-substrate interface, with surface concentrations decreasing with dose, approaching solid solubility limits at high doses. Implanted AlGaN/GaN maintained higher electron mobility than GaN, retaining a high-mobility 2DEG layer, though mobility decreased with increasing dose. Ti/Al ohmic contacts yielded specific resistances as low as 6.4 × 10^−8^ Ω·cm^2^ for GaN and ~10^−6^ Ω·cm^2^ for AlGaN, with sheet resistance tunable by dose.

**Figure 1:**
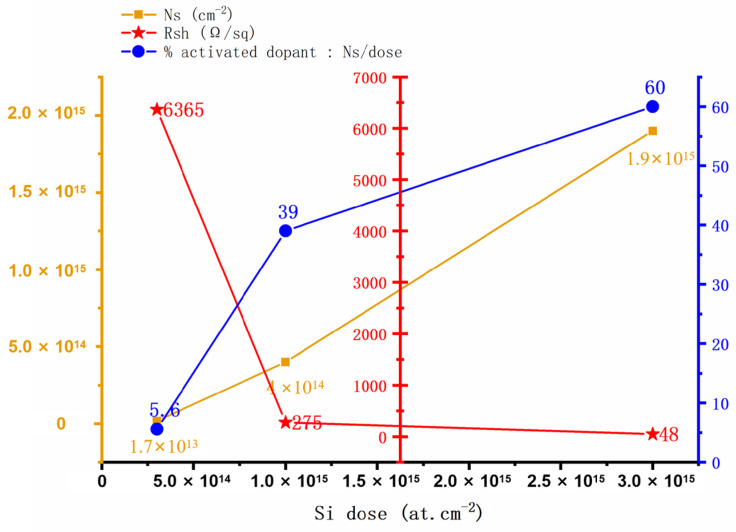
The dependence of GaN layer electrical properties on implanted Si dose was investigated. Samples were subjected to post-implantation annealing at 1100 °C for 1 h in N_2_ ambient, followed by four-probe resistance measurements using Ti (10 nm)/Au (30 nm)/Al (200 nm) contact structures. Adapted from [20].

N-type doping can be induced by Si ions which replace a portion of the Ga atoms in the GaN lattice [20]. Following implantation activation, mobile electrons are generated through Si ion dissociation, thereby establishing a conductive pathway within the semiconductor material. Figure 1 presents the electrical characteristics of GaN layers as a function of implanted Si dose after rapid thermal annealing at 1100 °C for 1 h under N_2_. It shows that with increasing Si dose from 3 × 10^14^ to 3 × 10^15^ cm^−2^, the sheet resistance decreases significantly from 6365 to 48 Ω/sq, and the percentage of activated Si increases from 5.6% to 60%, indicating that higher doses lead to lower resistive layers and higher activation rates. A higher injection dose and greater activation level can result in increased carrier concentration. By managing the injection dose and activation conditions of Si ions, the doping concentration and conductivity of GaN can be modulated effectively.

Due to the thermal decomposition limitation of GaN at elevated temperatures, dopant activation annealing of implanted species is constrained [21]. Elevating the substrate temperature effectively suppresses excessive Si ion diffusion during implantation, thereby achieving improved precision in ion implantation profiles. The Si depth distributions of samples implanted at RT and 500 °C (before/after 1200 °C, 5—min annealing), simulated by TRIM and measured by secondary-ion mass spectrometry (SIMS). The 500 °C implantation depth profile matches RT’s, so 500 °C does not affect Si ion distribution. Also, for both, annealing at 1200 °C does not change the Si depth profile, meaning Si ions do not diffuse drastically at this temperature. This approach simultaneously minimizes lattice disorder through reduced thermal budget and enhances dopant activation efficiency by promoting defect annealing kinetics. Additionally, Si ion implantation offers greater versatility in selective region doping compared to doping methods such as epitaxial growth and thermal diffusion.

According to Y. Jiang et al.’s work [17], Si ion implantation plays a crucial role in enhancing electron tunneling efficiency and facilitating the formation of ultra-low-resistance ohmic contacts on non-recessed i-InAlN/GaN heterostructures. Specifically, a thin Si interlayer (approximately 1–2 nm) is introduced, which enables the formation of a thin yet heavily doped n-type InAlN layer through Si doping; this significantly improves the tunneling transport of electrons by increasing the carrier density in the intrinsic InAlN barrier layer. Moreover, during high-temperature annealing, the in-diffused Si acts as a “catalyst” that promotes the decomposition of GaN, while the in-diffused Ti reacts with GaN—thermodynamically, this reaction (Ti (s) + GaN (s) ⇌ TiN (s) + Ga (s)) leads to the formation of TiN inclusions that penetrate through the entire InAlN barrier layer into the GaN channel. These TiN inclusions provide a direct contact pathway with the 2DEG, offering an additional route for electron injection into the channel. Furthermore, the synergistic effect of the tunneling mechanism (dominant below 900 °C, without TiN inclusions) and the spike mechanism (dominant above 900 °C, driven by TiN inclusions) alternately lowers the contact resistance, ultimately contributing to the achievement of an ultra-low contact resistance of 0.11 Ω·mm (*ρ*_c_ = 2.62 × 10^−7^ Ω·cm^2^).

### 3.2. Low-Resistance Ohmic Contact Using Ion Implantation Technique

Since the early 1990s, D. Qiao et al. [22] had suggested that direct injection of Si ions could significantly reduce the ohmic contact resistance. This is the first time that Si-ion implantation has been proposed for GaN devices. Si implantation was performed at 40 keV (direct) or 120 keV (through AlN) with 1 × 10^16^ cm^−2^, annealed at 1150 °C for 30 s in N_2_, achieving ~0.25 Ω·mm. In the following research on the regulation of device ohmic contact, the device ohmic contact is continuously reduced by updating the structure and innovating the failure mechanism. A comprehensive summary of these process innovations is presented below.

**Figure 2:**
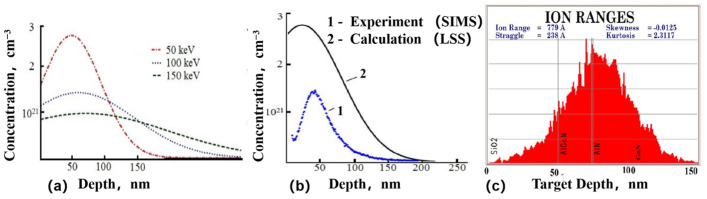
(**a**) The profiles of the concentration of Si^+^ with energies of 50, 100 and 150 keV using the LSS theory, the implantation dose is 10^15^ cm^−2^. (**b**) Experimental and calculated profiles of Si^+^ with energies of 50 keV and dose of 10^15^ cm^−2^. (**c**) Calculation of the maximum Si^+^ distribution in AlGaN/GaN with a 50 nm thick SiO_2_ film. Adapted from [28].

In 2004, H. Yu et al. [23] utilized a pressure fast thermal annealing technique to achieve doping activation and prepare ultra-low resistance ohmic contacts for Si ion-implanted GaN. Si was implanted in semi-insulating GaN at 100 keV with doses ranging from 5 × 10^14^ to 1.5 × 10^16^ cm^−2^, followed by rapid thermal annealing at 1500 °C with 100 bar N_2_ overpressure for 1 min, and Ti/Al/Ni/Au stack showed contact resistances of 0.07 Ω·mm (as-deposited) and 0.02 Ω·mm (subsequently annealed at 870 °C for 30 s). SIMS measurements showed that the as-implanted dopant distribution had a full width at half-maximum of 90 nm with a peak concentration of ~4 × 10^20^ cm^−3^, and after activation annealing, the peak broadened by 20 nm. Subsequently, in 2005, H. Yu et al. [24] developed an ultra-high-temperature (1500 °C) fast thermal annealing technique to activate Si dopants injected in the source and drain regions at 30 keV and 60 keV with 1.5 × 10^15^ cm^−2^ at 200 °C. The implanted GaN device with nonalloyed ohmic contacts demonstrates comparable device performance characteristics, achieving a contact resistance of 0.4 Ω·mm^2^, a maximum current density of 730 mA/mm^2^, and frequency response metrics of *f*_T_ = 26 GHz and *f*_max_ = 62 GHz. When operated under continuous wave (CW) bias conditions, the device delivers a peak power density of 3.4 W/mm^2^ on sapphire substrates. These preliminary results highlight the feasibility and reproducibility of incorporating ion implantation into GaN-based device processing. F. Recht et al. [8] (2006) conducted silicon ion implantation at room temperature in the source and drain regions and activation in a metal–organic chemical vapor deposition (MOCVD) system at a low activation annealing temperature (~1260 °C) for 30 s in N_2_/NH_3_. The experimental results demonstrate that the ion-implanted non-alloyed ohmic contact achieves a contact resistance of 0.96 Ω·mm^2^ at the channel region, with notable power performance characteristics including an output power density of 5 W/cm^2^, a power gain of 11.7 dB, and a power gain efficiency of 58%. These measurements were conducted at a frequency of 4 GHz under a drain bias voltage of 30 V. This study introduces a novel approach for preparing non-alloyed ohmic contacts in AlGaN/GaN HEMTs. In 2009, M. Placidi et al. [25] observed that samples with a SiO_2_ protective layer exhibited a more consistent *ρ*_c_ value of about 10^−5^ Ω·cm^2^ compared to samples without a protective layer, which also showed lower ρ_c_ values after metal alloying but with poorer reproducibility. However, the challenge arises from the difficulty of removing these protective layers after annealing, posing a hurdle for Si ion implantation. Simultaneously, T. Shiino et al. [26] suggested that dual-energy injection (80 keV with a fluence of 1.01 × 10^15^ cm^−2^ and 30 keV with a fluence of 1.6 × 10^14^ cm^−2^), followed by annealing at 1200 °C for 2 min in N_2_ atmosphere using a Si_3_N_4_ encapsulant, can create a high concentration of silicon atoms near the surface, increasing the carrier concentration in the n-type layer surface and reducing contact resistance. SRIM simulations and SIMS measurements show that it creates profiles with high Si concentrations near the surface and at the AlGaN/GaN interface. Furthermore, dual-energy injection diminishes the resistance of the n-type layer, thereby decreasing contact resistance. By employing single-energy injection, they achieved a reduction in contact resistance from 5.7 × 10^−7^ Ω·cm^2^ to 1.4 × 10^−7^ Ω·cm^2^. In the following year, C. Nguyen et al. [27] conducted tests on contact resistance after annealing at 1100 °C, 1150 °C, 1200 °C, and 1250 °C, revealing that the contact resistance of the injected samples reached record-low values during annealing at 1200 °C. Subsequent testing also indicated significantly low contact resistance of the injected samples during annealing. Moreover, in 2020, D. Fedorov et al. [28] used 50 keV (and 100 keV, 150 keV for calculations) with 10^15^ cm^−2^, annealed at 1250 °C for 1 min in N_2_ with SiO_2_/Si_3_N_4_, reducing resistance to 0.8 Ω·mm. The maximum concentration is at ~50 nm depth, implantation through a 50 nm SiO_2_ film shifts the peak to 25 nm (matching 2DEG depth), while a 100 nm film keeps the peak in the SiO_2_ (Figure 2).

J. Gallagher et al. [29] reported on selective area n-type doping in semi-insulating, C-doped GaN samples using conventional rapid thermal annealing (RTA) combined with 30 atm N_2_ overpressure annealing for activation. They tested the silicon activation in the implanted regions using Circular Transmission Line Model (CTLM) measurements. Linear and circular photoconductivity switches (PCSS) in non-implanted regions were employed as test instruments to compare the activation of implanted silicon dopants caused by the formation of N vacancies. The resulting leakage paths were isolated, while damage and decomposition were observed during the annealing process. Optimal contact resistivities as low as 1 × 10^−6^ Ω·cm^2^ were achieved at around 1060 °C while maintaining breakdown in unimplanted areas. The findings of this study serve as a valuable reference for exploring and optimizing doping and annealing processes for GaN materials, offering new ideas and possibilities for enhancing device performance and stability.

**Figure 3:**
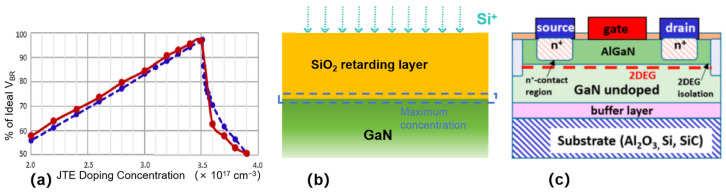
(**a**) Doping profile comparison between V_BR_ and JTE structures in single zone JTE devices: Edge concentration gradient control differences between uniform diffusion (dashed lines) and ion implantation (solid lines). Adapted from [30]. (**b**) Schematic illustration of Si^+^ injection with SiO_2_ as retarding layer. Adapted from [31]. (**c**) Schema of AlGaN/GaN HEMT. Adapted from [33].

Si ions are injected into the p^+^ layer to introduce additional free electrons, reducing the resistance of the p^+^ layer by M. Shurrab [30]. This injection is performed using an ion implantation apparatus, where an accelerated ion beam is injected into the p^+^ layer. For example, with a p^+^ epilayer doping concentration of 2 × 10^18^ cm^−3^ and thickness of 0.4 µm, a desired JTE doping concentration of 3 × 10^17^ cm^−3^ requires a donor concentration of 1.7 × 10^18^ cm^−3^ (corresponding to an n-type dose of 6.8 × 10^13^ cm^−2^). By controlling such dose and energy parameters—with energies tested at 20 keV to 240 keV and total doses reaching 6.74 × 10^13^ cm^−2^—the concentration and distribution of Si ions are adjusted to achieve the desired doping in the p^−^ JTE region. This forms low-resistance p^−^ JTE regions in the p^+^ layer, enabling low-resistance contact and high V_BR_, with the maximum V_BR_ reaching 96% of the ideal value (Figure 3a).

A. Kozubal et al. explored Si^+^ implantation into GaN for ohmic contact formation [31], with a 350 nm SiO_2_ retarding layer proving optimal for achieving a quasi-uniform Si dopant profile through a single implantation of 250 keV ions (Figure 3b). Effective fluences of 5.4 × 10^15^ cm^−2^ and 1.1 × 10^16^ cm^−2^ were employed, where the higher fluence increased point defect concentration and dislocation density but significantly enhanced electrical performance. Activation annealing at temperatures ranging from 900 to 1100 °C played a crucial role, with higher temperatures reducing point defects—most notably at 1100 °C—leading to a sheet resistance as low as 37.5 ± 1.8 Ω/sq., a substantial improvement over the value at 900 °C. The choice of encapsulation layer was also critical: a newly deposited 200 nm SiO_2_ layer, applied after removing the retarding layer, outperformed the “old SiO_2_” configuration that retained the retarding layer, boosting net doping by two orders of magnitude from 2.3 × 10^17^ cm^−3^ to 2.3 × 10^19^ cm^−3^ and avoiding carrier trapping from GaN surface degradation. These combined parameters resulted in excellent ohmic contact properties, including a specific contact resistance as low as 0.03 ± 0.02 Ω·mm for the 1.1 × 10^16^ cm^−2^ fluence with 1100 °C annealing, validating the approach’s value for GaN-based electronic and optoelectronic devices.

A. Chanuel et al. [32] proposed an access technique to scale the Ka-Band GaN HEMT. This study compared the off-state characteristics of silicon implant-assisted contacts with those of conventional recessed titanium/aluminum-based ohmic contacts. The transistor with the source/drain extension via the silicon implant has low contact resistance, the RC value is as low as 0.4 Ω·mm, and the implanted layer is 67 Ω/sq. Transistors incorporating source/drain extension regions not only exhibit excellent ohmic contact characteristics but also achieve high breakdown voltage (BV) levels in compact device dimensions through enhanced electrostatic control, thereby enabling high-frequency operation with optimal reliability. Comprehensive investigations on gate-source/drain terminals and channel length revealed a novel failure mechanism involving subchannel current flow beneath the gate region, leading to source-drain breakdown. In contrast, traditional titanium alloy contacts exhibit breakdown behavior dominated by punch-through effects. The experimental data demonstrates that silicon-injection-assisted contact technology shows significant practical applicability in GaN HEMTs, enabling innovative approaches to enhance device performance through optimized electrical characteristics, thermal reliability, and integration density while expanding operational efficiency boundaries.

Si ion implantation can create highly doped n-type source/drain regions on lightly doped p-type GaN, as well as selectively increase the doping level underneath the contact on n-type GaN [33] to achieve ohmic contact without the need for subsequent metallization annealing (Figure 3c). However, Si ion implantation for ohmic contacting techniques in GaN devices may face some difficulties in practice, as the injection leads to high impurity doping and lattice damage, which affects the electrical properties of the contact. In addition, the necessity to protect the sample surface during high temperature annealing (1000–1200 °C) in order to achieve electrical activation of the n-type Si dopant is also a factor to be considered.

## 4. Electrical Field Modulation of GaN-Based Power Device Using Ion Implantation Technique

GaN lateral device termination structures are strategically designed to simultaneously control electric field distribution and optimize current spreading through dual mechanisms [34]: electrostatic shielding to suppress local field peaks and reduced lateral resistance for enhanced current transport efficiency. This integrated approach not only minimizes crosstalk interference and thermal stress by regulating current density gradients but also extends device reliability under static and dynamic operating conditions. By balancing both electrostatic and thermal constraints, these structures enable higher power density and improved carrier mobility characteristics, making them critical for advancing GaN-based applications in power electronics, RF systems, and optoelectronics while addressing the challenges of device breakdown and performance degradation in high-stress environments [35]. This section systematically investigates the functional roles of F, N, and Ar ions in GaN lateral devices through comparative analysis of their respective contributions to device termination characteristics.

### 4.1. F Ion Implantation

The role of F ion implantation in GaN enhancement-mode devices is a key technology aimed at optimizing device performance. Fluorine has extremely high electronegativity and tends to gain electrons to be negatively charged. When implanted into GaN, its electronegativity can change the local charge distribution of the material, adjust carrier concentration and mobility, and optimize interface properties, thereby improving device performance. This section will discuss in detail the role, principles, advantages and future development directions of F ion implantation in GaN enhancement-mode devices. F ion implantation introduces negatively charged fluorine ions into specific areas of GaN devices to change their electrical properties and electric field distribution [36]. The introduction of F ions can effectively modulate the electric field distribution inside the device, especially in the edge region of the junction. By rationally designing the position and dose of F ion implantation, the electric field can be weakened or evenly distributed, thereby improving the breakdown voltage and reliability of the device. F ion implantation can change the carrier concentration and mobility in GaN devices, thereby adjusting the conductive performance and speed of the device. This helps reduce on-resistance and increase switching speed, resulting in higher power density and efficiency. The introduction of F ions can also improve the interface quality between GaN devices and other materials, reduce interface state density and surface roughness [37]. The modulation of threshold voltage in GaN devices by F ion implantation can be classified into three categories, which are material property modulation, electric field regulation and contact optimization.

**Figure 4:**
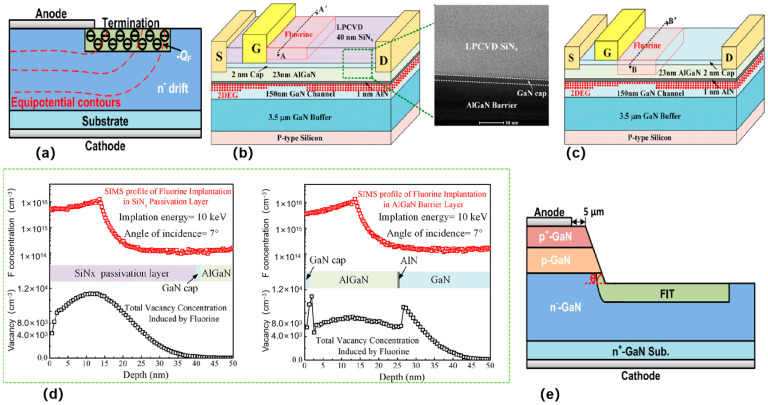
(**a**) Terminated-SBD with edge-terminated charges suppresses high fields near the main junction. Adapted from [40]. (**b**) Schematic diagram of FPL HEMT. Adapted from [39]. (**c**) Schematic diagram of FBL HEMT. Adapted from [39]. (**d**) The fluorine ion concentration profile form A-A′ (left) and B-B′ (right), measured via SIMS, and the vacancy concentration profile, simulated by TRIM. Adapted from [39]. (**e**) Schematic diagram of the vertical GaN PiN diode with a single-zone bevel structure. Adapted from [40].

In 2019, S. Han et al. [38] has realized a vertical gallium nitride Schottky rectifier with high blocking voltage and low forward voltage drop by F-ion implantation and a low-temperature thermal annealing process. As shown in Figure 4a, F ion injection can introduce negative fixed charge and effectively suppress the electric field concentration in the junction region, thus reducing the reverse leakage current and increasing the blocking voltage. A low-temperature thermal annealing process restores the injection-induced damage and further reduces the reverse leakage current. C. Yang et al. [39] presents an AlGaN/GaN HEMT structure with high breakdown voltage and low dynamic on-resistance by F ion injection in a thick SiN_x_ passivation layer (FPL HEMT) (Figure 4b). FBL HEMT can be seen in Figure 4c. The advantages of fluorine ion implantation in SiN_x_ passivation layer over AlGaN blocking layer (FBL HEMT) are significant. FPL HEMT outperforms FBL HEMT by suppressing dynamic current collapse (*R*_ON,D_ rises only 23% at 100 V vs. 98% for FBL), achieving higher BV (803 V vs. 746 V) via passivation-layer-engineered E-field modulation, and minimizing 2DEG perturbation through spatial isolation of fluorine-induced defects in the thick SiN_x_ layer, all while maintaining process compatibility and avoiding parasitic capacitance. When fluorine ions are implanted with identical energy and dose, the FPL and FBL exhibit nearly identical measured peak positions (from the surface) and maximum concentrations in their fluorine profiles (Figure 4d). Fluorine-induced vacancies in the thin AlGaN barrier layer extend to the 2DEG channel, while those in the thick SiN_x_ passivation layer stay within the layer and far from the 2DEG, reducing impact on the channel and suppressing characteristic degradation. S. Han et al. [38] applied post-implantation annealing (PIA) at 450 °C in N_2_ for 10 min with a SiN_x_ as a prevention layer which can hinder the possible out—diffusion of F ions after multi-energy F ion implantation. F ion profiles measured by SIMS indicate that it would be a significant measure to protect F ions from escaping during PIA.

Y. Liu et al. [40] (2020) presented a novel fluorine-implanted termination (FIT) structure for optimizing the vertical GaN PiN diode by introducing negatively charged F ions to mitigate electric field crowding at the junction edge, achieving a higher BV. This innovative approach involved comprehensive Technology Computer-Aided Design (TCAD) simulations to explore key FIT parameters, such as F ion dosage, FIT thickness and width, angle of inclination, and more, to unveil their impact on BV. The research revealed that the presence of negatively charged F ions effectively disperses the electric field surrounding the main junction, thus preventing premature breakdown. Additionally, the quantity of F ions in the FIT area emerged as a crucial factor influencing the BV of the vertical GaN PiN diode. Utilizing a bevel structure (Figure 4e), the study demonstrated the confinement of the maximum electric field within the block, leading to improved BV within the low F ion dosage range. Furthermore, through partial single-step ion implantation, a cone-shaped distribution of F ion dosage in the FIT area was achieved, which expanding the FIT dose window and enhancing the BV of the diode. The optimized FIT structure not only mitigates electric field crowding but also improves the overall BV performance of the vertical GaN PiN diode. By carefully controlling the parameters of the FIT, such as F ion dosage, bevel angle, double-zone FIT, etc., the diode’s reliability and efficiency can be significantly enhanced. This research opens up new avenues for designing high-performance vertical GaN PiN diodes with improved breakdown voltage characteristics. The findings highlight the potential of FIT structures in enhancing the stability and reliability of power electronic devices, paving the way for further advancements in GaN device technology.

**Figure 5:**
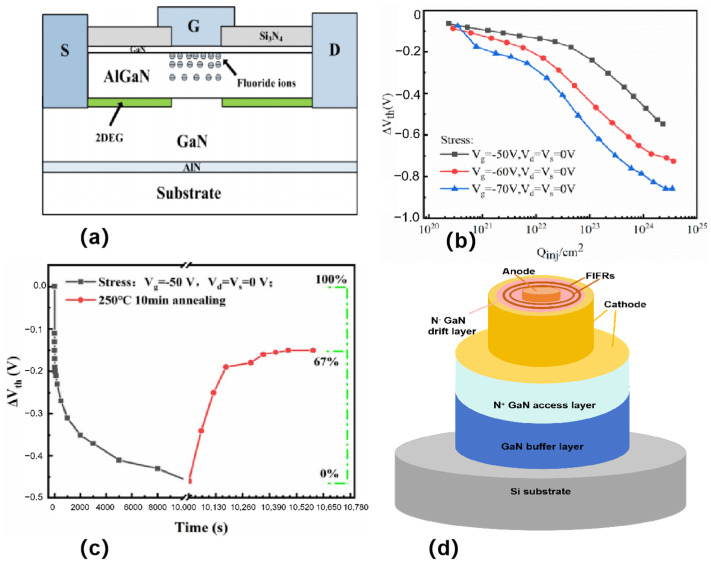
(**a**) Schematic cross section of E-mode HEMTs fabricated by fluoride-based implantation technology [41]. (**b**) ΔV_th_ versus Q_inj_ relationships in E-mode HEMTs under varying reverse gate biases, with Q_inj_ representing charge accumulation during reverse bias operation [41]. (**c**) ΔV_th_ shifts in E-mode HEMTs under reverse gate bias during temperature annealing at 250 °C [41]. Images have been obtained with permission from IEEE Publishing. (**d**) Schematic cross section of a Schottky barrier diode (SBD) with fluorine-implanted field rings (FIFRs). Adapted from [42].

Defects can be introduced via F-ion implantation [41], and these defects lead to a decrease in the threshold voltage at high electric fields (Figure 5a). As shown in Figure 5b, it can be observed experimentally that the threshold voltage of the E-type HEMT is negatively shifted after a period of time under high reverse gate bias conditions. During high reverse gate bias, a certain number of neutral traps are generated in the AlGaN barrier layer, which are located near the channel. After the annealing experiment, the electrons in the channel will gain enough energy and tunnel into the neutral traps, resulting in negatively charged neutral traps, which reduces the number of electrons in the channel, thus causing a positive shift in the threshold voltage (Figure 5c). Improved device reliability and gate control capability.

J. Chen et al. [42] (2021) introduced negative regions that are called terminal rings for F ion injection (Figure 5d). The presence of the termination rings moves the high reverse electric field from the top Schottky contact into the semiconductor body, thereby reducing the concentration of the electric field at the edge of the electrodes and increasing the breakdown voltage of the device. The specific method to achieve F ion injection is to inject F ions into GaN crystals through an ion implantation device. In the experiments, the energy level of F ion injection was 25 keV and the dose was 2 × 10^14^ cm^−2^. After injection, the samples were annealed to allow the F ions to form a stable structure in the crystal. The width of the terminal ring was 2 μm, and the difference between the inner radius and the radius of the anode was 1 μm. By introducing the terminal ring for F-ion injection, the leakage current at high reverse voltages can be suppressed effectively, and the breakdown voltage of the device can be improved. Figure 5d shows the SBD with FIFRs and sidewall cathodes which exhibits a low *V*_on_ of 0.54 V. This approach improves the performance of GaN-on-Si quasi-vertical Schottky barrier diodes with lower turn-on voltage. This study has obtained a lower reverse current density, which is beneficial for increasing the reverse breakdown voltage. Therefore, compared with other studies, the SBD with FIFRs has a significant advantage in the overall performance of turn on voltage and breakdown voltage.

By gradually decreasing the F-ion injection region from gate to drain in Graded Fluoride ion implantation Terminal HEMT(GFT HEMT), the electric field distribution in the drift region can be regulated more effectively, which can significantly improve the BV [43]. The electric field distribution with GFT HEMT is more uniform and has a higher breakdown voltage (955 V) than the conventional F-ion implantation terminal (CON HEMT). And by injecting F ions into a thick encapsulation layer, physical damage to the AlGaN material can be avoided and the adverse effect on the 2DEG mobility can be reduced.

**Figure 6:**
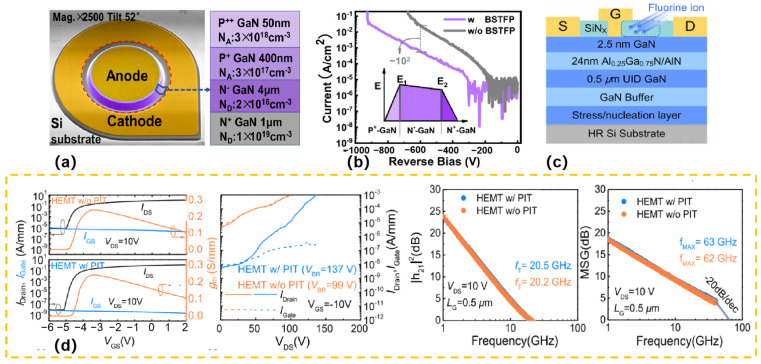
(**a**) A pseudo-colored scanning electron microscopy (SEM) image of a BSTFP diode is presented, accompanied by a schematic representation of the device’s epitaxial structure [44]; (**b**) Reverse I-V characteristics of devices incorporating BSTFP and reference devices without BSTFP are presented [44]; (**c**) Structure schematic of the fabricated AlGaN/GaN-on-Si HEMTs [45]; (**d**) The physical properties of HEMTs without PIT process and with PIT process are compared from left to right: Transfer I-V characteristics; Three terminal OFF-state breakdown; Current gain; Maximum stable gain versus frequency [45]. Images have been obtained with permission from IEEE Publishing.

Combining inclined side walls and self-aligning F plasma treatment (BSTFP)can enhance the performance of GaN-on-Si quasi-vertical PiN diodes [44] (Figure 6a). This diode not only achieves an excellent breakdown voltage (930 V), but also has extremely low reverse leakage current. Moreover, the dual diode exhibits a low specific on-resistance (0.43 mΩ·cm^2^), a high ON/OFF (*I*_ON_/*I*_OFF_) and a superior Baliga Merit Value (2.01 GW/cm^2^). XPS and Kelvin Probe Force Microscopy confirmed that F ions were present and decreased the device’s surface potential, which in turn decreased the electric field peak and suppressed the leakage current (Figure 6b). These results highlight the huge potential of GaN on Si PiN diodes for power applications. By optimizing structural design and surface treatment, this research provides an important reference for improving device performance and stability.

F ions can also inject onto the SiN_x_ passivation layer [45] through the passivation implanted termination (PIT) technique (Figure 6c). Negative charges are introduced, thus mitigating the electric field peaks at the gate edge. Figure 6d presents the transfer I-V characteristics, three-terminal OFF-state breakdown voltage, and small-signal RF performances of GaN-on-Si HEMTs with and without the passivation implanted termination (PIT) process. The transfer I-V characteristics show that the OFF-state leakage current of the PIT-HEMT is three orders of magnitude lower than that of the conventional HEMT, attributed to the termination of the peak electric field at the gate edge. The three-terminal OFF-state breakdown measurements indicate that the breakdown voltage of the PIT-HEMT is 137 V, higher than 99 V of the conventional one, with the passivation termination structure improving breakdown characteristics without introducing additional parasitic capacitances. For small-signal RF performances, the conventional HEMTs have a *f*_T_ of 20.2 GHz and a *f*_MAX_ of 62 GHz, while the PIT-HEMTs exhibit *f*_T_ of 20.5 GHz and *f*_MAX_ of 63 GHz, confirming no parasitic capacitance is introduced by fluorine ion implantation on SiN_X_. This reduces the OFF-state leakage current, improves the drain current dispersion, increases the threshold voltage, and raises the breakdown voltage. However, the negative fixed charge generated by the F ion injection on the barrier layer causes significant intrinsic carrier losses, thus limiting the current capacity and power density of GaN-based HEMTs for RF applications.

F ions treatment has the ability to reduce dark current. K. Zhou et al. [46] (2023) highlighted the growing interest in UV photodetectors (UVPDs) based on AlGaN/GaN HEMTs, which has become a hot research topic. The apparatus generates ultra-high responsivity and high photocurrent by using two-dimensional electron gas (2DEG) formed on an AlGaN/GaN interface, which is made from C_4_F_8_ gas. In this study, the researchers successfully reduced the dark current of the device and effectively suppressed the persistent photoconductivity (PPC) effect. Specifically, through in situ fluorine plasma treatment, the dark current is reduced by about 3 orders of magnitude, the injected F ions effectively suppress the PPC effect, and the response speed is also significantly improved, with rise/fall times of 69/90 ms, respectively. This research result provides a simple and cost-effective strategy for manufacturing high-performance UVPDs based on AlGaN/GaN HEMTs. And it is expected to promote further development in the field of UV photodetectors.

### 4.2. N Ion Implantation

X. Guo et al. [47] (2021) introduced a novel nitrogen implanted guard ring (GR) technique for 600 V quasi-vertical GaN-on-Si Schottky barrier diodes (SBD). The GR parameters are designed and researched by means of TCAD simulation. Research has shown that N-ion-injected GR can significantly reduce the electric field peaks of GaN SBDs, and the minimum electric field peaks can be obtained when the width of the GR is about 1–2 μm. The I-V characteristics indicate that the breakdown voltage of the SBD can be improved by the GR injected by N ions. Ultimately, by utilizing a 4.4 μm thick GaN drift layer, the seven precision-engineered GRs significantly increased the breakdown voltage of GaN SBDs to approximately 600 V, while maintaining excellent on-state properties. These include an *I*_ON_/*I*_OFF_ ratio of up to 10, an ideality factor of 1.06, an ON-voltage of 0.64 V (at 1 A/cm^2^), and a specific on-resistance (*R*_ON,sp_) of 1.40 mΩ·cm^2^, setting a new performance benchmark. At 175 °C, *R*_ON,sp_ of 7 GRs increased slightly to 1.86 mΩ·cm^2^, maintaining excellent electrical properties even at a backward bias of 380 V, which demonstrating the high temperature operation ability of N-injected GR. X. Guo et al. [48] used triple-energy nitrogen ion injection to form protection rings to mitigate the effect of electric field concentration at the anode metal edges. Ion implantation works by creating a high concentration of nitrogen ions in the edge region. Ion implantation leads to the filling of electron traps in the n-GaN drift layer, reducing electron trapping, altering the electric field distribution, thereby reducing the peak electric field at the edges and increasing the breakdown voltage of the devices.

Y. Wang et al. (2023) [49] introduced a novel GaN guard ring (GR) edge termination design achieved through selective regional nitrogen implantation of a p-GaN layer. This termination, formed by p-GaN rings separated by implanted semi-insulating regions, simplifies fabrication with only a single implantation step. This approach enhances process flexibility and eliminates the need for precise depth control. The number and spacing of these rings determine the avalanche breakdown voltage of a vertical GaN p-n diode, with the 16-ring structure achieving 88% of the theoretical parallel plane limit. In contrast, T. Nelson et al. [50] (2022) proposed a Hybrid Junction Termination Extension with a GR superimposed over the JTE, creating regions with alternating depths of implantation. By integrating the charge distribution in the terminal region and fitting it to that of the reference terminal, an optimization strategy for the design geometry is presented. Simulation results show that the breakdown voltage is 92% to 95% higher than that of the ideal parallel plane limit. Increasing the number of rings improves the match with the reference terminal’s charge distribution, leading to a better approximation of the breakdown voltage response to changes in p-layer doping concentration and thickness. This design approach enables TCAD optimization of high-voltage device terminal geometry with minimal computational cost.

Similarly to the self-aligned fluorine plasma treatment of the GaN-on-Si diode sidewalls performed by F. Jia et al. [44], J. Priesol et al. [51] demonstrated that lateral isolation in semi-vertical GaN power diodes was effectively achieved through nitrogen implantation, creating a distinct boundary between the active device area and the N-implanted region. During cross-sectional SEM-EBIC measurements performed at room temperature using a field emission gun SEM LEO 1550 with an e-beam incidence angle of ~45°, a sharp drop in the EBIC signal was observed at this boundary, which could be clearly identified in both low and high-leakage current samples. This significant reduction in the EBIC signal, resulting from the insulating effect induced by nitrogen ions, serves as direct evidence that the N-ion implantation successfully fulfills the role of lateral isolation, effectively separating the active region from the N-implanted region and ensuring the proper electrical isolation required for the device’s functionality.

The conventional JTE method is difficult to achieve in GaN devices due to the challenging side-defined p-type doping in nitrides. However, Y. Duan et al. (2023) [52] successfully utilized nitrogen ion implantation to selectively compensate dopants during the growth of p-GaN epitaxial layers, leading to the development of JTE structures. Specifically, by nitrogen ion injection, fully and partially compensated regions can be created within the p^+^ region. By adjusting the injected dose and energy, the concentration of defects induced by ion injection can be calculated as a function of depth in each region. To achieve smaller thicknesses in the second and third regions, the injected energy can be increased to enhance the extent of the injection. This approach enables the realization of a three-region stepped JTE structure. By reducing the thickness of the p-layer in each region, the electric field can be effectively dispersed, thereby decreasing the peak electric field in the device and enhancing the reverse performance of the vertical GaN diode.

### 4.3. Ar Ion Implantation

In GaN-on-Si devices, the mismatch of lattice constants and thermal expansion coefficients between GaN and Si presents significant challenges for the growth of crack-free thick GaN drift layers on Si substrates [53]. At the same time, accurate doping of GaN drift layers are critical for a good compromise between *R*_on_ and BV. However, impurities such as O, Si, and C are usually accidentally doped, which act as donors or acceptors in GaN. These impurities typically result in fluctuations in the net carrier concentration within the GaN drift layer. The electric field crowding effect is an obstacle to achieve high BV. To mitigate this effect, ion-implantation based edge terminations (ETs), such as argon injection termination (ArIT), have been proposed and applied to GaN- and SiC-based power devices grown on free-supported substrates. Argon is an inert gas with extremely inactive chemical properties and hardly reacts with other substances. In GaN devices, when argon ions are implanted to form a high-resistance region, its inertness ensures that it will not chemically react with the GaN material, avoiding introducing additional impurities or changing the original chemical structure of the material. The authors of [54] investigate disorder accumulation in GaN implanted with 150 keV Ar^+^ ions, examining the effects of ion dose from 3 × 10^14^ to 3 × 10^16^ cm^−2^ and implantation temperature from room temperature to 1000 °C using RBS/C and SIMS. They identify two disordered regions (surface and bulk damage peaks) with distinct transition doses, finds that increasing implantation temperature reduces surface peak damage but has less impact on the bulk peak, observes a “reverse annealing” behavior in the bulk peak within a specific dose range from 8 × 10^14^ to 4 × 10^15^ cm^−2^ attributed to secondary defects, and nots that high doses suppress this reverse regime, with damage decreasing monotonically with temperature. Few reports on vertical GaN-on-Si diodes with effective ETs have been reported so far. The principle of argon injection termination is to form a high-resistance region by injecting argon ions in the edge region of the device. This high-resistance region effectively mitigates the electric field crowding effect, thereby increasing the breakdown voltage [55]. When the device is in reverse bias, the electric field becomes concentrated near the argon injection region, thereby reducing the peak electric field near the junction. This reduction in the electric field helps to increase the breakdown voltage of the device. Argon injection termination can be achieved by using argon ion injection technique during the fabrication process, which creates a high-resistance region in the edge region of the GaN-on-Si device. This technique is effective in improving the performance of vertical GaN-on-Si diodes, including increasing the breakdown voltage and reducing the electric field crowding effect.

In 2021, X. Guo et al. [56] formed an ArIT in GaN material by ion-implantation during the preparation of an ArIT-SBD (gallium nitride Schottky diode). Argon ion implantation was performed with an energy of 50 keV and a dose of 1 × 10^16^ cm^−2^, using the Schottky contact metal as the implantation mask. The thermal annealing was conducted at 550 °C in N_2_ ambient for 5 min. Damage-induced traps in the ion injection region can capture the injected electrons and form negative charges. These negative charges can partially deplete the drift layer in the GaN material and change the potential distribution, thereby increasing the BV of the device. In addition, the traps in the ion injection region can change the electric field distribution, mitigating the strength of the electric field in the vicinity of the Schottky junction, thereby reducing the occurrence of tunneling currents. Additionally, X. Guo et al. [57] proposed that in vertical GaN-on-Si SBDs without edge termination, the current leakage characteristics sequentially undergo hot-electron field emission, variable-range hopping (VRH), and trap-assisted tunnelling conductivity mechanisms as the reverse bias gradually increases. The leakage and breakdown mechanisms are limited by the edge electric field crowding effect. In contrast, for vertical GaN-on-Si SBDs with ArIT (ArIT-SBDs), at low reverse bias, the electron conductivity follows the space charge limited conductivity (SCLC) model, which is limited by damage-induced traps in the injected GaN. As the reverse bias increases to the point where breakdown occurs, VRH and SCLC dominate the leakage mechanisms of ArIT-SBD, which originate from the intrinsic traps in GaN grown on Si. At low reverse bias, the leakage of ArIT-SBD grows rapidly and the breakdown voltage performance is enhanced, which is attributed to the charging of damage-induced traps in the injected GaN, provides a useful guide for the design of injection termination for high voltage power devices.

## 5. Conclusions

This review systematically evaluates the pivotal role of ion implantation techniques in advancing GaN-based device performance. A brief summary is presented in Table 1. Extensive research has demonstrated that ion implantation significantly enhances electrical characteristics and lateral termination engineering, thereby establishing itself as a cornerstone technology for GaN device optimization. These investigations have not only provided critical technical validation but also laid theoretical frameworks for device design, fabrication, and performance improvement, effectively paving the way for technological breakthroughs in this field.

Notwithstanding these advancements, critical challenges persist in the reliability assessment of GaN devices subjected to ion implantation processes. Specifically, temperature stability, including hot carrier effects, thermal degradation, and trap-related phenomena, remains a major obstacle. Additionally, there exists a critical need for further investigations into the microstructural mechanisms governing ion-doping interactions within GaN materials. Addressing these fundamental issues is essential for achieving next-generation device performance improvements. Future research should focus on developing in situ characterization techniques to monitor implantation dynamics and explore novel dopant engineering strategies, which present promising avenues for future exploration.

## Figures and Tables

**Table 1:** The influence of N, F, Si, and Ar ion implantation on GaN devices.

Ion	Effects	Performance	Properties
N ions	Form high-resistance regions for electrical isolation; High-dose implantation enhances leakage suppression and voltage withstand	Lower leakage current; Enhance breakdown voltage; Improve high-temperature stability; Provide isolation without extra layers/roughness	High electronegativity, reactivity; Low post-implantation annealing temperature; Maintain low defect density; Enhance thermal/electrical stability
F ions	Introduce defects that affect threshold voltage under high electric fields; Mitigate electric field crowding at junction edges and gate edges; Reduce interface state density;	Enhance breakdown voltage; Low forward voltage drop and turn-on voltage; Decrease dynamic on-resistance; Improve ON/OFF ratio; Reduce dark current	High electronegativity leads to negative charging; Reduce 2DEG perturbation; Engineer the electric field distribution
Si ions	Increase carrier concentration; Improve crystal quality; Higher fluence increases defects	Lower on-state resistance; Reduce contact resistance; Enhance saturation drain current; Boost transconductance	Atomic radius of Si is closer to that of Ga; Excellent chemical compatibility with GaN
Ar ions	Form high-resistance regions; Induce damage-induced traps; Weaken the electric field near the Schottky junction	Increase the breakdown voltage; Alleviate electric field crowding; Capture injected electrons; Reduce tunneling current; Improve leakage characteristics	Inactive chemical properties; Preventing introduction of additional impurities
